# Behavioral Convergence: Implications for Mathematical Models of Sexually Transmitted Infection Transmission

**DOI:** 10.1093/infdis/jiu431

**Published:** 2014-12-01

**Authors:** Sevgi O. Aral, Helen Ward

**Affiliations:** 1 Division of STD Prevention, National Center for HIV/AIDS, Viral Hepatitis, STD, and TB Prevention,Centers for Disease Control and Prevention, Atlanta, Georgia; 2 Department of Infectious Disease Epidemiology, School of Public Health, Imperial College London, United Kingdom

**Keywords:** sex work, mathematical models, sexually transmitted infections, sexual behavior

## Abstract

Recent trends in the behaviors of some groups with high sexual activity and of the general population in some countries suggest that sexual behavior profiles of high and low sexual activity categories may be converging and may call into question the assumptions around sexual mixing that are built into theoretical models of sexually transmitted infections (STIs)/human immunodeficiency virus (HIV) transmission dynamics. One category of high sexual activity, sex work, has been undergoing modification in many societies, becoming more acceptable, more dispersed, and larger in volume in some societies and shrinking in others. Concurrent with changes in the characteristics of sex work, the accumulating data on the sexual behaviors of the general population suggest a shift toward those of sex workers, including large numbers of sex partners and short-duration partnerships. The closing of the gap between behaviors associated with high and low sexual activity may have important implications for theories of sexual structure and models of transmission dynamics for STIs, including HIV infection.

Theoretical depictions of sexual structure used in mathematical models of sexually transmitted infection (STI) and human immunodeficiency virus (HIV) transmission dynamics often compartmentalize individual members of populations into categories of high and low sexual activity and make assumptions regarding the extent of sexual mixing within and between these categories [1]. These categories have always been simplifications of reality but are used to improve our understanding of transmission dynamics for conditions in which random population mixing would not explain persistence [2]. Recent trends in the behaviors of some high-sexual-activity groups and of the general population in some countries suggest that sexual behavior profiles of high and low sexual activity categories may be converging and may call into question the assumptions around sexual mixing that are built into theoretical models of STI/HIV transmission dynamics.

High-sexual-activity groups are variously defined, but characteristics include numbers of sex partners, frequency of sexual contact, types of sexual contact, and the resulting dense sex networks. In many models, high-sexual-activity groups comprising women are often conflated with sex workers. This can lead to circular reasoning, such that high risk is assumed to be sex work and, therefore, sex work and associated practices are assumed to be high risk. However, while in many populations sex workers and their clients have indeed been at increased risk of acquisition and transmission of sexually transmitted infections (STIs) and HIV, in others they have not [3, 4].

Sex workers are not the only subgroup used to structure models; in many cultural contexts, other characteristics of high-risk groups, such as same-sex relationships and diversity of sexual repertoire have become more widely reported and accepted. What was once seen as deviance has been increasingly normalized or referred to as sexual diversity, and this tendency may blur what previously appeared to be clear boundaries [5]. Concurrently, in the general population, sexual behaviors are reported that may resemble those of sex workers, such as very-short-term partnerships with few social or relationship ties. These changes may indicate a convergence between the behaviors associated with high and low sexual activity or may reveal the inadequacies of binary simplifications. As the boundaries between categories (ie, high or low) sexual activity categories get blurred, depictions of societal sexual structure in conceptual models may need to be modified.

In this perspective article, we briefly summarize demographic trends and outline changes in levels of acceptance and frequency of sex work. Next, we discuss changes in sexual behaviors of the general population in countries such as the United States and Great Britain. Finally, we list the dominant characteristics of commercial sex networks and discuss how sex networks of the general population might increasingly display similar attributes. We conclude with a brief discussion of the implications of the changes mentioned above for theoretical models of sexual structure.

## DEMOGRAPHIC TRENDS IN SEXUAL INSTITUTIONS

Over the past 2 decades, patterns of marriage, divorce, and cohabitation have changed. Together with the aging of the population, this has led to many more adults living alone, which has been described as an enduring feature of the contemporary developed world [6]. In the European Union, for example, almost 1 in 5 households (18%) are single adults aged <65 years [7]; in Sweden, 47% of all households have just 1 resident, and in the United States, this proportion is 28% [6]. This is reflected in a fall in average household size across the Organisation for Economic Co-operation and Development (OECD) countries, from 2.8 to 2.6 individuals, between the 1980s and 2000s [8].

In many countries, marriage rates have been declining [9]. In the United States, the marriage rate per 1000 population declined from 8.2 in 2000 to 7.6 in 2005 and 6.8 in 2010. A similar pattern is observed in European countries [10]. Marriage rates in OECD countries fell from >8 per 1000 in 1970 to 5 per 1000 in 2009, while average divorce rate doubled to 2.4 per 1000 [8]. Marriage rates are declining in Asia, as well [11]. Moreover, age at marriage is increasing for both men and women globally [10, 11]. Concurrent with declining marriage rates, divorce rates have risen in many countries, including countries of the European Union. The world's highest divorce rates are observed in Eastern Europe: Russia, Moldova, Belarus, and Ukraine [10, 11]. As a result of these demographic trends, people spend a longer period of their adult lives outside of marriage (both before marriage and after divorce) than ever before. Marital mixing patterns have also changed. High earning and educated women are more likely to be married than they were a couple of decades ago, and in the United Kingdom and United States, there has been a 3-fold increase in the proportion of male–female partnerships in which the woman is at least 5 years older than the man [12].

These trends in household and family institutions result from a multiplicity of social determinants and constitute adoptive responses to fundamental changes taking place in drivers such as women's education level and employment, greater independence, technology, economy, globalization, and capitalization [13].

## THE SEX INDUSTRY

It is not only civil institutions that have changed significantly during the past couple of decades. Sex work and its impact on health has undergone considerable modification [14]. While sex work is highly variable across different contexts, in many societies important shifts have occurred in some salient parameters that define this occupation.

Sex work is heterogeneous with respect to the demographic characteristics and socioeconomic background of sex workers, socioeconomic background of clients, sexual services offered, patterns and venues of solicitation, venues where sex takes place, whether sex work is practiced full- or part-time, and, whether from a life-course perspective, sex work is transitional or represents a lifelong career [15]. In many contexts, some predominant characteristics of sex work have been changing over the past 3 decades. There appears to have been a shift to a more diverse and fluid workforce: in earlier decades, sex workers were often from poor socioeconomic backgrounds, and now, women from a wide range of socioeconomic backgrounds and education levels go into sex work. In many countries, women with higher degrees and professional training may be found engaging in sex work. For example, in the former Soviet Union, physicians, professors, and nurses are engaged in sex work [16–18], while in the United Kingdom, a number of women returned to sex work after obtaining professional qualifications in other fields [19]. Sex work has always been combined with other roles, whether as homemakers or to supplement poorly paid work, but this appears to have become more widespread [20]. Observation of these changes may be in part an artifact of the increased surveillance and research on sex work as a result of concerns about HIV, public order, or migration. Poor and marginalized women were always the most visible as street- or bar-based workers; more middle class and educated women have always been involved in sex work but may have been hidden [21].

Closely related to part-time practice is the issue of transiency, a growing feature of much employment in recent decades, which has been described in terms of a growing precariat population [22, 23]. Women, and men, combine different types of work, formal and informal, which may include going into and out of sex work for shorter periods. Also, in the past, sex work was predominantly spatially segregated, leading to the concept of red-light districts. Now, in many societies, sex work tends to be highly spatially dispersed and mobile, in part because of mobile communication technology [13]. Frequent moves across different neighborhoods maximize the exposure of different areas to sex work. All these changes tend to blur the boundaries and minimize the social distance between sex workers and the general population, contributing to the greater acceptance and wider spread of sex work in some cases. When women who live on one's own street engage in sex work, it becomes more difficult to stereotype and discriminate against sex workers [24]. In other cases, this is considered socially disruptive, and new methods of controlling sex work are being introduced, such as the widespread criminalization of clients in Europe.

Another change involves the apparent expansion of the volume and range of forms of what can broadly be termed transactional sex. Although prostitution, in the past, was often defined in terms of a formal exchange of money for sex, other types of trade have been common but less obvious. By including exchange for other things of value into the definitions of sex work/transactional sex, the size of the high-risk category has expanded, and the distinction between commercial and other relational forms has blurred [25, 26]. In Zimbabwe, the economic crisis was associated with a dispersal of sex work from bars to the community, and an increase in the exchange of sex for goods and other support as cash became less valuable in the face of hyperinflation [27].

The organization of sex work has changed with these evolving characteristics. Currently, the organization of sex work takes many diverse forms, which have often brought with them greater choice and a shift away from greater hierarchical dependency to greater autonomy.

Most of the evidence supporting the generalizations above is qualitative, observational, and anecdotal. The phenomena under discussion involve sex work, sex work Web sites, sex partner recruitment sites, sex tourism, female and child sex tourism, child sexual abuse, casual sex, transactional sex, and group sex. Systematically compiled empirical evidence on representative samples of groups involved in these practices is scarce. In fact, formulation of specific hypotheses based on qualitative observations may encourage the conduct of quantitative empirical studies in the future.

Sex work and sex tourism have become an important means of supporting national economies in many economically struggling countries. Many Eastern European cities are now destination sites for sex tourism [14]. Kiev and Odessa, Ukraine, Riga, Latvia, Prague, Czech Republic, and Krakow, Poland, all attract predominantly male, short-term migration streams from other areas, including the former Soviet Union, Europe, North America, Middle East, and Africa. Upon arrival in Kiev airport, passengers are handed maps of Kiev with advertisements for sex services on the side, complete with photographs and phone numbers. Liberal attitudes, a preference for foreigners, and difficult economic circumstances, combined with a desire for available consumer products, motivate women and make sexual connections between local women and visiting men easier and more attractive (Aral and St Lawrence, unpublished reports).

Western Internet introduction and marriage sites are worldwide businesses that make many millions and facilitate international sex tourism. A Google search for “Russian and Ukrainian Women” yields >150 “new beautiful ladies every week” [28].

Initiation into sex work is often accomplished with the help of friends who are already in the business. This was the pattern observed in Moscow, St. Petersburg, and Saratov, Russia, and in Tallinn, Estonia, between 2000 and 2010 [16–18, 24]. The recruitment process would be initiated by controllers or pimps and completed through sex workers, who brought their friends into the business. Elsewhere, women actively seek work and approach friends and others already in the business. This pattern appears to be an indication of the widespread nature and greater acceptability of sex work, a result of economic pressures and behavioral contagion.

At present, in many contexts, sex work seems to be expanding. It is less stigmatized than earlier, it pays more than alternative career choices, and it is often backed by enabling technology and international business. In North Dakota, United States and Alberta, Canada, the recent booming oil riches have brought with them exploding volumes of commercial sex activity. Social determinants play an important role in this context. While some determinants, such as unemployment and poverty, create necessities, others, such as inequality and consumer economy, create desires.

In other contexts, sex work appears to be shrinking. According to data collected in the General Social Survey conducted by the National Opinion Center at the University of Chicago (Chicago, IL), fewer men say they have ever paid for sex—or been paid for it—compared with data from a few decades ago. In a series of surveys between 1991 and 1996, nearly 17% of men said they had ever paid for (or received payment for) sex. This proportion fell to 13.2% between 2006 and 2012. In 2013, the number hit the lowest point—9.1%— since the question was first asked [29]. These proportions appear to be shifting with the generations. Older men are considerably more likely to say they have bought sex at some point, while younger men are less likely to report doing so, compared with men of the same ages a few decades ago. Anecdotal evidence is concurrent with this picture. In the United States, men often report they prefer to have girlfriends to visiting sex workers, even if they financially support the women functioning as girlfriends. Despite convergence of findings in data collected through different sources, using a variety of methods, it is important to use caution in interpreting these results. Over time, interpretations of different sexual experiences evolve, the language used to describe particular types of sexual connections changes, and levels of reporting bias involved in self-reporting of sexual experience is never the same.

The most recent and most solid data on proportions reporting exchange of sex for payment come from the NATSAL III, in Great Britain [30]. While the percentage of men reporting such exchange increased from 2.1% to 4.3% between NATSAL I and NATSAL II, it appeared to stabilize at 4% in NATSAL III in 2010. Despite the high quality of this survey, it remains difficult to assess the role of changes in both willingness to report and definitions of sex work.

## SEXUAL BEHAVIORS IN THE GENERAL POPULATION

The accumulating data on the sexual behaviors of the general population suggest a shift toward those more traditionally associated with commercial sex. Characteristics of sex work behaviors include large numbers of sex partners, not much discrimination in choice of sex partners, short period between the time 2 people meet each other and the time they engage in sex, short time spent during the sexual encounter, lack or short duration of any social links between sex partners, short duration of gaps between consecutive sex partners and consecutive sexual encounters, tendency for both partners to recruit each other for sex, and receipt of compensation for sex. All these characteristics appear to be on the rise in the sexual behaviors of the general population, particularly among younger cohorts. Moreover, of late, behaviors of women have been changing more rapidly than behaviors of men [30].

The domain of sexual relationships in everyday lives of members of the general population appears to be large and perhaps expanding. Older people are increasingly sexually active into later years. In one 2012 study, many older women (mean age, 67 years) reported that sex gets better with age; half of the women were sexually active, with arousal, lubrication, and orgasm into old age, despite low libido in one third of the participants [31]. The changing and less restrictive norms regarding expected sexual mixing across age groups may be a factor affecting sexual experiences of older women.

Among younger cohorts, a so-called hook-up culture also reflects more-permissive sexual mixing norms across age groups, as well as other social categories and roles [32]. Over the past few decades, hook-up behavior, defined as sexual activity between uncommitted individuals, has become pervasive among young adults. The long window of sexually mature but prereproductive life, the greater number of years that people live in single households, the evolved neural correlates of sexual behavior, and the greater acceptance of the sexualization of youth all contribute to the emergence and stabilization of the hook-up culture [32]. The social spaces in which members of the general population initiate and engage in sexual behaviors are numerous and may be expanding. Hook-up behaviors on college campuses have been reported by many authors [32].

Recent cohort analyses of data from the United States [33] and Britain [34] both suggest that more-recent cohorts tend to initiate sex earlier, have greater numbers of sex partners over their lifetime, report greater frequency of same-sex sexual behaviors, and have a larger sexual repertoire. In both societies, changes among men happened earlier than changes among women; in both societies, most recently, women are becoming more like men. The proportion of men who report ≥10 sex partners exceeds that of women in all societies at all times. However, the gender gap in this parameter seems to be shrinking; more young women report this behavior than older women. In all economically developed countries, sexual repertoires are expanding in the general population. Proportions who report practicing oral and anal sex have been increasing in successive generations; increases in oral sex happened with the baby boomers, and increases in anal sex happened more recently with generation X and the millennials. Whether this trend continues to accelerate in response to the expansion of pornography on the Internet remains to be seen.

## CHANGES IN SEXUAL NETWORK PATTERNS

The changes in the sexual behaviors of the general population discussed above, almost by definition, led to the spread of sex networks that resemble commercial sex networks. Sex networks of sex workers and their clients have several identifiable properties, including mutual nonmonogamy, short-duration partnerships, short gaps between partnerships, and high mobility and turnover among individuals participating in such networks. The mobility and turnover levels are important because they increase levels of sexual mixing among subpopulations in important ways. Mutual nonmonogamy is expected to increase in the general population when the gap between women's numbers of sex partners and those of men becomes smaller. Hook-up culture increases the frequency of short-term links in important ways. Current patterns of behavior increase the prevalence of commercial-sex-like links in the general population.

## CONCLUSION

The evidence reviewed here suggests that, in many societies, sex work is becoming more diverse and transient as the sexual behaviors and networks of the general population increasingly resemble and overlap those of sex workers and their clients. Moreover, sex tourism, which has expanded in volume in recent years, sexually links the high-sexual-activity groups across different countries.

These hypotheses, if supported through systematically collected quantitative data, may have important implications for theories of sexual structure and models of transmission dynamics for STIs and HIV [34]. Assumptions regarding the differentiation between sexual activity classes, patterns of sexual mixing, and the extent to which societies can be considered sexually closed populations may need to be reexamined. Models are most useful for policy makers when they are as simple as possible and only as complex as necessary [35]. To explore whether essential structures of models such as activity classes and mixing need to be refined in the light of shifts in population behaviors requires a close relationship between anthropologists, behavioral scientists, and epidemic modelers.
